# DAILY STRESS PROCESSES, PHYSICAL ACTIVITY, AND SLEEP IN OLDER ADULTHOOD

**DOI:** 10.1093/geroni/igad104.1925

**Published:** 2023-12-21

**Authors:** Kate Leger, Susan Charles, Colette Brown, Karen Fingerman

**Affiliations:** University of Kentucky, Lexington, Kentucky, United States; University of California, Irvine, Irvine, California, United States; UCI, Irvine, California, United States; The University of Texas at Austin, Austin, Texas, United States

## Abstract

Older adults who engage in healthy behaviors such as being physically active and sleeping well report lower levels of stress. Less is known about the links between exposure and reactivity to stressful events in daily life and their relationship with physical activity and sleep. The current study examined within-person associations between daily physical activity, sleep, and exposure and affective reactivity to naturally occurring interpersonal stressors. Older adults (N = 180) from the Daily Experiences and Well-being Study completed ecological momentary assessments (EMAs) every 3 hours for 5-6 days where they reported negative affect throughout the day and interpersonal tensions at the end of the day. They also reported their nightly sleep quality and wore Actical accelerometers to capture physical activity. Older adults reported greater numbers of stressors on days when they spent less time being sedentary and engaged in more light physical activity. On days when older adults experienced more stressors, they reported higher levels of negative affect, but this association was attenuated when they were more physically active that day. Additionally, older adults reported lower sleep quality the night after they reported higher numbers of stressors. These findings underscore the importance of assessing physical activity, sleep and stressful events in daily life and have implications for both physical and psychological well-being.

